# Bis(2-carb­oxy­quinolinium) hexa­chlorido­stan­nate(IV) dihydrate

**DOI:** 10.1107/S2414314624008265

**Published:** 2024-08-30

**Authors:** Mohamed Siradj Eddine Benhamada, Tarek Benlatreche, Rochdi Ghallab, George Dénès, Hocine Merazig

**Affiliations:** a Environmental Molecular and Structural Chemistry Research Unit University of Constantine-1 25000 Constantine Algeria; b Ecole Nationale Polytechnique de Constantine (ENPC) Laboratoire de Technologie des Matériaux Avancés Algeria; c Laboratory of Solid State Chemistry and Mössbauer Spectroscopy, Department of Chemistry and Biochemistry, Concordia University, 7141 Sherbrooke St. West, Monreal, H4B 1R6 QC, Canada; Vienna University of Technology, Austria

**Keywords:** crystal structure, [SnCl_6_]^2–^ anion, hydrogen-bonding, π–π stacking

## Abstract

In the hydrated title salt, (C_10_H_8_NO_2_)_2_[SnCl_6_]·2H_2_O, the tin(IV) atom is located about a center of inversion. In the crystal structure, the organic cation, the octa­hedral inorganic anion and the water mol­ecule of crystallization inter­act through O—H⋯O, N—H⋯O and O—H⋯Cl hydrogen bonds, supplemented by weak π–π stacking between neighboring cations, and C—Cl⋯π inter­actions.

## Abstract 2

In the title compound, the 2-carb­oxy­quinolinium cation, the [SnCl_6_]^2–^ anion (site symmetry 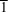) and the water mol­ecule of crystallization are held together by hydrogen bonds, π–π stacking and C—Cl⋯π inter­actions.

## Graphical abstract

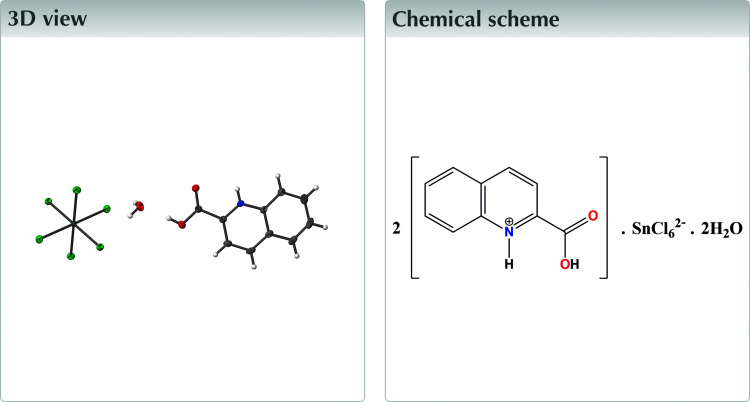

## Structure description

The crystal structure determination of the title compound, (I), was undertaken as part of studies of organic–inorganic hybrid materials, which may exhibit various inter­esting physical properties such as dielectric characteristics (Hajlaoui *et al.*, 2013 ▸).

**Figure 1:**
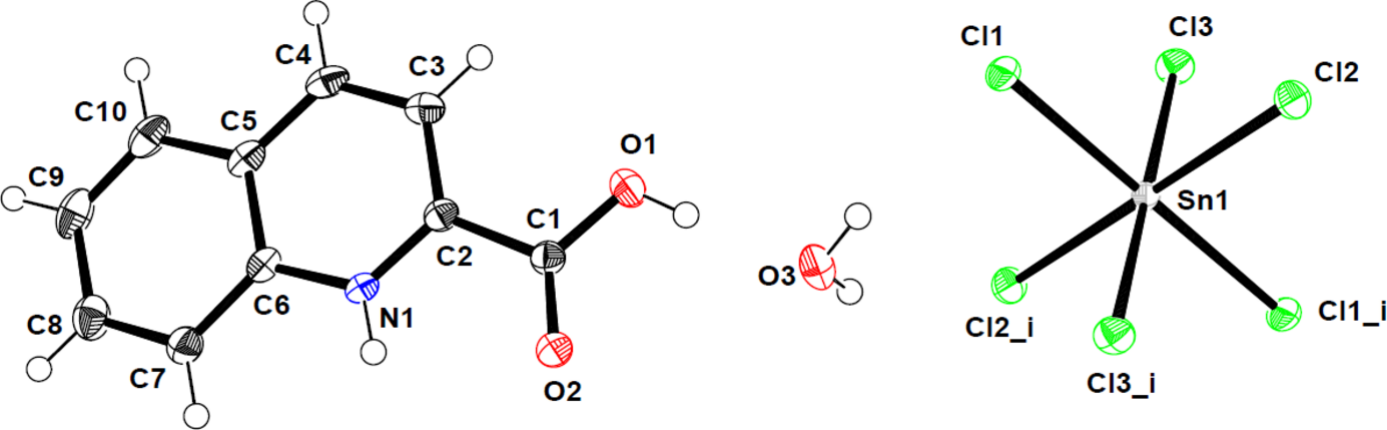
View of the mol­ecular entities in (I) with the atom-numbering scheme. Displacement ellipsoids for non-H atoms are drawn at the 50% probability level. [Symmetry code: (i) −*x*, −*y*, −*z*].

The asymmetric unit of (I) comprises half of an octa­hedral [SnCl_6_]^2–^ anion (the whole anion being completed by inversion symmetry), one 2-carb­oxy­quinolinium cation, and a water mol­ecule of crystallization (Fig. 1 ▸).

The Sn^IV^ atom is coordinated by six chlorine atoms, forming a slightly distorted octa­hedron. The lengths of the Sn—Cl bonds in the hexa­chlorido­stannate(IV) anion range from 2.4180 (3) to 2.4406 (3) Å and the Cl—Sn—Cl bond angles deviate only by approximately 1° from ideal values, similar to those reported in the literature (Ghallab *et al.*, 2020 ▸; Rademeyer, 2004 ▸; Billing *et al.*, 2007 ▸).

In the 2-carb­oxy­quinolinium cation, the C—C bond lengths range from 1.364 (2) to 1.5029 (15) Å and the C—N bond lengths are 1.3282 (14) and 1.3649 (17) Å; the angles vary between 115.14 (10) (N1—C2—C1) and 126.71 (11)° (O1—C—O2). These values are similar compared with related cations with protonated aromatic N atoms (Gelmboldt *et al.*, 2007 ▸; Smith *et al.*, 2004 ▸, 2008 ▸). The cation in (I) is not planar, as indicated by a dihedral angle between the quinoline ring and the carb­oxy group of 11.61 (9)°.

**Figure 2:**
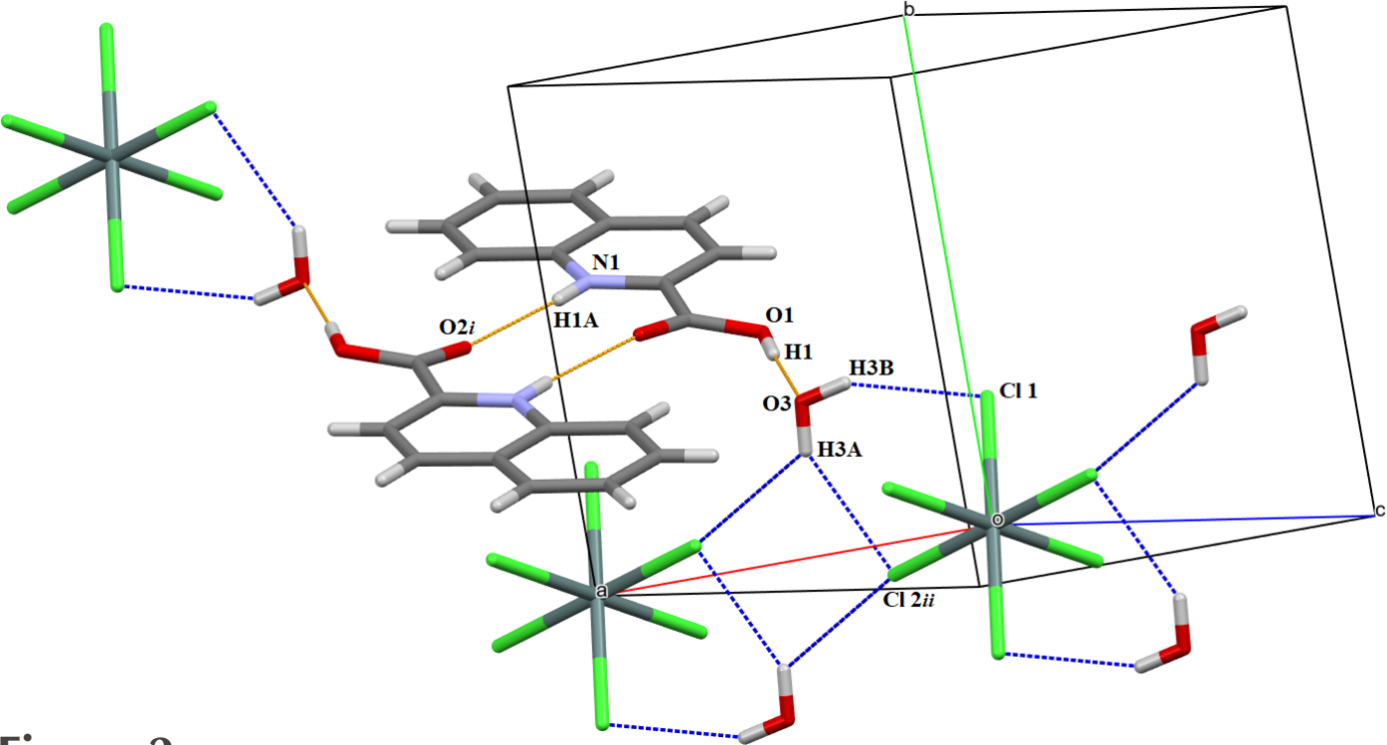
O—H⋯O, N—H⋯O and O—H⋯Cl hydrogen-bonding inter­actions in the crystal structure of (I) indicated by dashed lines. Symmetry codes refer to Table 1 ▸.

**Figure 3:**
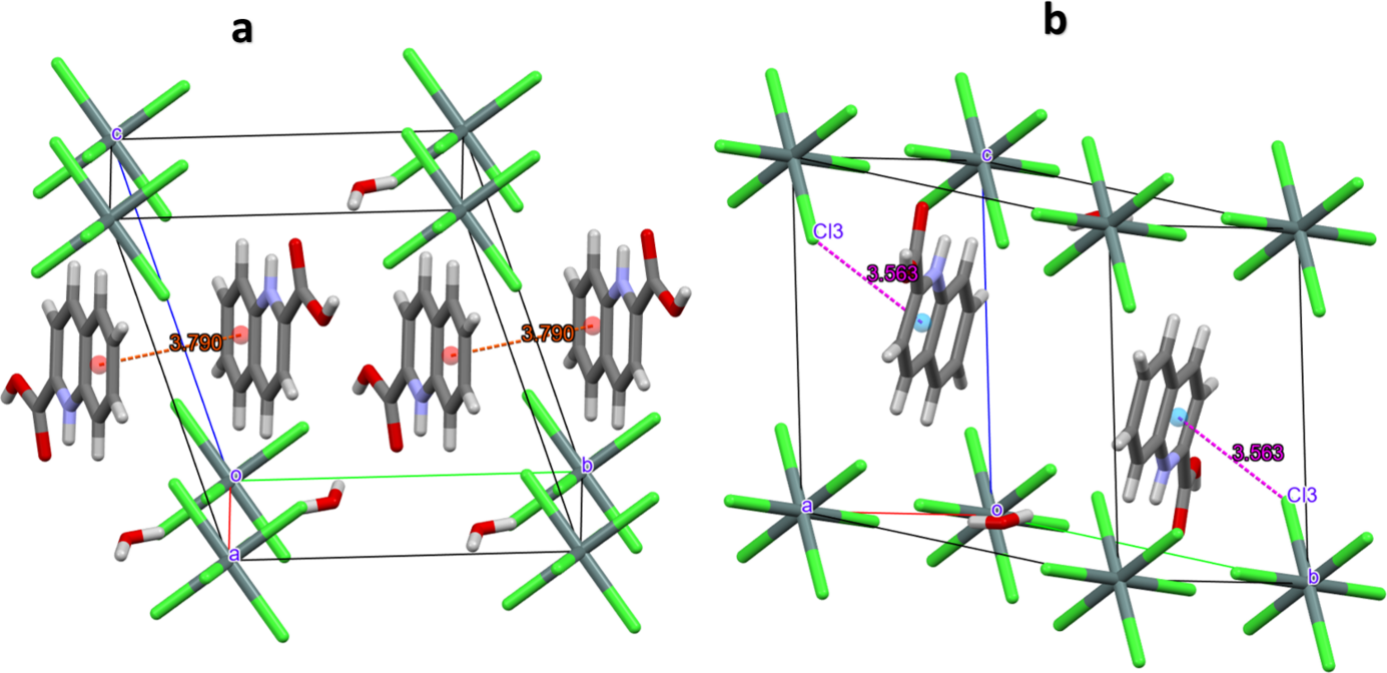
(*a*) π–π stacking inter­actions and (*b*) Cl⋯π inter­actions in the crystal structure of (I).

Apart from Coulombic forces, the cohesion in the crystal structure is ensured by classical hydrogen bonds between the carboxyl group as donor and the water mol­ecule as acceptor groups, and by inter­actions between the protonated quinoline N atom and the non-protonated O atom of the carboxyl group of a neighboring mol­ecule. Further inter­actions involve the water mol­ecule and the hexa­chlorido­stannate anion (Table 1 ▸, Fig. 2 ▸). There are also π–π stacking inter­actions between neighboring cations [3.7898 (8) Å, slippage 1.678 Å; Fig. 3 ▸*a*] and Cl⋯π inter­actions [3.5633 (6) Å; Fig. 3 ▸*b*].

## Synthesis and crystallization

Tin(II) chloride dihydrate (SnCl_2_·2H_2_O) was mixed with quinaldic acid (C_10_H_7_NO_2_) in a 1:2 molar ratio, along with a few drops of hydro­chloric acid in distilled water. The mixture was refluxed for one h at 343 K. After two weeks of slow solvent evaporation at room temperature, colorless single crystals suitable for X-ray analysis were obtained.

## Refinement

Crystal data, data collection, and structure refinement details are summarized in Table 2 ▸. The H atom bound to the N atom was refined freely. 14 reflections were omitted from the refinement because they were obstructed from the beam stop.

## Supplementary Material

Crystal structure: contains datablock(s) I. DOI: 10.1107/S2414314624008265/wm4218sup1.cif

Structure factors: contains datablock(s) I. DOI: 10.1107/S2414314624008265/wm4218Isup4.hkl

CCDC reference: 2378953

Additional supporting information:  crystallographic information; 3D view; checkCIF report

## Figures and Tables

**Table 1:** Hydrogen-bond geometry (Å, °)

*D*—H⋯*A*	*D*—H	H⋯*A*	*D*⋯*A*	*D*—H⋯*A*
O1—H1⋯O3	0.82	1.75	2.5684 (14)	176
N1—H1*A*⋯O2^i^	0.832 (17)	2.028 (17)	2.8348 (13)	163.4 (15)
O3—H3*A*⋯Cl1	0.85	2.46	3.2048 (11)	146
O3—H3*B*⋯Cl2^ii^	0.85	2.61	3.3708 (11)	150

Symmetry codes: (i) 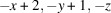; (ii) 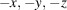.

**Table 2:** Experimental details

Crystal data	
Chemical formula	(C_10_H_8_NO_2_)[SnCl_6_]·2H_2_O
*M*_r_	715.77
Crystal system, space group	Triclinic, *P* 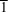
Temperature (K)	296
*a*, *b*, *c* (Å)	8.3220 (4), 9.2704 (4), 9.4248 (4)
α, β, γ (°)	108.101 (2), 99.515 (2), 99.749 (2)
*V* (Å^3^)	662.46 (5)
*Z*	1
Radiation type	Mo *K*α
μ (mm^−1^)	1.61
Crystal size (mm)	0.10 × 0.09 × 0.08

Data collection	
Diffractometer	Bruker APEXII CCD
Absorption correction	Multi-scan (*SADABS*; Krause *et al.*, 2015 ▸)
*T*_min_, *T*_max_	0.851, 0.879
No. of measured, independent and observed [*I* > 2σ(*I*)] reflections	19467, 3262, 3199
*R*_int_	0.015
(sin θ/λ)_max_ (Å^−1^)	0.667

Refinement	
*R*[*F*^2^ > 2σ(*F*^2^)], *wR*(*F*^2^), *S*	0.013, 0.033, 1.08
No. of reflections	3262
No. of parameters	168
H-atom treatment	H atoms treated by a mixture of independent and constrained refinement
Δρ_max_, Δρ_min_ (e Å^−3^)	0.41, −0.25

Computer programs: *APEX2* and *SAINT* (Bruker, 2013 ▸), *OLEX2.solve* (Bourhis *et al.*, 2015 ▸), *SHELXL* (Sheldrick, 2015 ▸), *OLEX2* (Dolomanov *et al.*, 2009 ▸) and *publCIF* (Westrip, 2010 ▸).

## full crystallographic data

### Bis(2-carboxyquinolinium) hexachloridostannate(IV) dihydrate Crystal data

**Table:**

(C_10_H_8_NO_2_)[SnCl_6_]·2H_2_O	*Z* = 1
*M**_r_* = 715.77	*F*(000) = 354
Triclinic, *P*1	*D*_x_ = 1.794 Mg m^−^^3^
*a* = 8.3220 (4) Å	Mo *K*α radiation, λ = 0.71073 Å
*b* = 9.2704 (4) Å	Cell parameters from 9611 reflections
*c* = 9.4248 (4) Å	θ = 3.7–48.2°
α = 108.101 (2)°	µ = 1.61 mm^−^^1^
β = 99.515 (2)°	*T* = 296 K
γ = 99.749 (2)°	Plate, yellow
*V* = 662.46 (5) Å^3^	0.10 × 0.09 × 0.08 mm

### Bis(2-carboxyquinolinium) hexachloridostannate(IV) dihydrate Data collection

**Table:**

Bruker APEXII CCD diffractometer	3199 reflections with *I* > 2σ(*I*)
φ and ω scans	*R*_int_ = 0.015
Absorption correction: multi-scan (SADABS; Krause *et al*., 2015)	θ_max_ = 28.3°, θ_min_ = 4.8°
*T*_min_ = 0.851, *T*_max_ = 0.879	*h* = −11→11
19467 measured reflections	*k* = −12→12
3262 independent reflections	*l* = −12→12

### Bis(2-carboxyquinolinium) hexachloridostannate(IV) dihydrate Refinement

**Table:**

Refinement on *F*^2^	0 restraints
Least-squares matrix: full	Hydrogen site location: mixed
*R*[*F*^2^ > 2σ(*F*^2^)] = 0.013	H atoms treated by a mixture of independent and constrained refinement
*wR*(*F*^2^) = 0.033	*w* = 1/[σ^2^(*F*_o_^2^) + (0.013*P*)^2^ + 0.3169*P*] where *P* = (*F*_o_^2^ + 2*F*_c_^2^)/3
*S* = 1.08	(Δ/σ)_max_ < 0.001
3262 reflections	Δρ_max_ = 0.41 e Å^−^^3^
168 parameters	Δρ_min_ = −0.25 e Å^−^^3^

### Bis(2-carboxyquinolinium) hexachloridostannate(IV) dihydrate Special details

**Table:**

Geometry. All esds (except the esd in the dihedral angle between two l.s. planes) are estimated using the full covariance matrix. The cell esds are taken into account individually in the estimation of esds in distances, angles and torsion angles; correlations between esds in cell parameters are only used when they are defined by crystal symmetry. An approximate (isotropic) treatment of cell esds is used for estimating esds involving l.s. planes.

### Bis(2-carboxyquinolinium) hexachloridostannate(IV) dihydrate Fractional atomic coordinates and isotropic or equivalent isotropic displacement parameters (Å^2^)

**Table:**

	*x*	*y*	*z*	*U*_iso_*/*U*_eq_	
Sn1	0.000000	0.000000	0.000000	0.01307 (3)	
Cl1	0.12549 (3)	0.26567 (3)	0.17700 (3)	0.01985 (6)	
Cl2	−0.27553 (3)	0.05974 (3)	−0.01450 (3)	0.02261 (6)	
Cl3	−0.03577 (4)	−0.08014 (3)	0.21592 (3)	0.02122 (6)	
O2	0.82893 (11)	0.48826 (10)	0.04894 (9)	0.02362 (17)	
O1	0.70770 (11)	0.47285 (12)	0.24138 (10)	0.02734 (19)	
H1	0.625567	0.421382	0.171718	0.041*	
O3	0.44565 (12)	0.30407 (12)	0.03220 (12)	0.0316 (2)	
H3A	0.358189	0.328438	0.058776	0.047*	
H3B	0.438485	0.207555	0.016123	0.047*	
N1	1.12611 (12)	0.62982 (11)	0.25392 (11)	0.01629 (17)	
C2	0.98812 (14)	0.60663 (12)	0.30529 (12)	0.0171 (2)	
C1	0.83151 (14)	0.51554 (13)	0.18355 (13)	0.0183 (2)	
C3	0.99422 (16)	0.66408 (14)	0.46178 (13)	0.0213 (2)	
H3	0.897184	0.649793	0.497561	0.026*	
C6	1.28072 (14)	0.70531 (13)	0.34788 (13)	0.0181 (2)	
C9	1.59120 (17)	0.85434 (15)	0.54706 (16)	0.0299 (3)	
H9	1.696466	0.902964	0.612341	0.036*	
C8	1.57523 (16)	0.79829 (15)	0.38631 (16)	0.0277 (3)	
H8	1.670263	0.812255	0.347661	0.033*	
C7	1.42258 (15)	0.72382 (14)	0.28635 (14)	0.0230 (2)	
H7	1.413118	0.686630	0.180899	0.028*	
C5	1.29308 (15)	0.76284 (13)	0.50879 (13)	0.0208 (2)	
C10	1.45388 (17)	0.83779 (15)	0.60721 (14)	0.0274 (3)	
H10	1.465775	0.875747	0.712974	0.033*	
C4	1.14626 (16)	0.74234 (14)	0.56255 (13)	0.0230 (2)	
H4	1.151849	0.782030	0.667260	0.028*	
H1A	1.119 (2)	0.5947 (19)	0.160 (2)	0.026 (4)*	

### Bis(2-carboxyquinolinium) hexachloridostannate(IV) dihydrate Atomic displacement parameters (Å^2^)

**Table:**

	*U*^11^	*U*^22^	*U*^33^	*U*^12^	*U*^13^	*U*^23^
Sn1	0.01189 (5)	0.01279 (5)	0.01377 (5)	0.00232 (3)	0.00262 (3)	0.00407 (4)
Cl1	0.02182 (13)	0.01429 (11)	0.01922 (12)	0.00029 (9)	0.00555 (10)	0.00173 (9)
Cl2	0.01509 (12)	0.02717 (14)	0.02324 (13)	0.00878 (10)	0.00250 (10)	0.00457 (11)
Cl3	0.02591 (13)	0.02106 (13)	0.01886 (12)	0.00433 (10)	0.00618 (10)	0.01006 (10)
O2	0.0205 (4)	0.0291 (4)	0.0164 (4)	0.0005 (3)	0.0033 (3)	0.0045 (3)
O1	0.0201 (4)	0.0358 (5)	0.0226 (4)	−0.0002 (4)	0.0071 (3)	0.0077 (4)
O3	0.0207 (4)	0.0325 (5)	0.0368 (5)	−0.0007 (4)	0.0106 (4)	0.0069 (4)
N1	0.0185 (4)	0.0158 (4)	0.0123 (4)	0.0036 (3)	0.0020 (3)	0.0028 (3)
C2	0.0197 (5)	0.0152 (5)	0.0164 (5)	0.0049 (4)	0.0035 (4)	0.0053 (4)
C1	0.0182 (5)	0.0175 (5)	0.0187 (5)	0.0047 (4)	0.0046 (4)	0.0051 (4)
C3	0.0257 (6)	0.0217 (5)	0.0175 (5)	0.0074 (4)	0.0075 (4)	0.0063 (4)
C6	0.0196 (5)	0.0150 (5)	0.0167 (5)	0.0038 (4)	0.0005 (4)	0.0037 (4)
C9	0.0239 (6)	0.0260 (6)	0.0301 (6)	0.0012 (5)	−0.0089 (5)	0.0069 (5)
C8	0.0201 (6)	0.0267 (6)	0.0330 (7)	0.0044 (5)	0.0023 (5)	0.0084 (5)
C7	0.0210 (6)	0.0235 (6)	0.0213 (5)	0.0036 (4)	0.0029 (4)	0.0051 (5)
C5	0.0258 (6)	0.0164 (5)	0.0164 (5)	0.0044 (4)	−0.0003 (4)	0.0037 (4)
C10	0.0310 (7)	0.0246 (6)	0.0188 (5)	0.0035 (5)	−0.0053 (5)	0.0042 (5)
C4	0.0326 (6)	0.0216 (5)	0.0132 (5)	0.0078 (5)	0.0035 (4)	0.0039 (4)

### Bis(2-carboxyquinolinium) hexachloridostannate(IV) dihydrate Geometric parameters (Å, º)

**Table:**

Sn1—Cl3	2.4180 (3)	C2—C1	1.5029 (15)
Sn1—Cl3^i^	2.4180 (3)	C3—C4	1.3758 (17)
Sn1—Cl1	2.4370 (3)	C3—H3	0.9300
Sn1—Cl1^i^	2.4370 (3)	C6—C7	1.4067 (17)
Sn1—Cl2^i^	2.4406 (3)	C6—C5	1.4220 (15)
Sn1—Cl2	2.4406 (3)	C9—C10	1.364 (2)
O2—C1	1.2099 (14)	C9—C8	1.4155 (19)
O1—C1	1.3026 (14)	C9—H9	0.9300
O1—H1	0.8200	C8—C7	1.3715 (17)
O3—H3A	0.8511	C8—H8	0.9300
O3—H3B	0.8496	C7—H7	0.9300
N1—C2	1.3282 (14)	C5—C4	1.4041 (18)
N1—C6	1.3649 (14)	C5—C10	1.4171 (17)
N1—H1A	0.832 (17)	C10—H10	0.9300
C2—C3	1.3927 (15)	C4—H4	0.9300
			
Cl3—Sn1—Cl3^i^	179.999 (12)	O1—C1—C2	112.24 (10)
Cl3—Sn1—Cl1	89.336 (10)	C4—C3—C2	118.86 (11)
Cl3^i^—Sn1—Cl1	90.665 (10)	C4—C3—H3	120.6
Cl3—Sn1—Cl1^i^	90.663 (10)	C2—C3—H3	120.6
Cl3^i^—Sn1—Cl1^i^	89.336 (10)	N1—C6—C7	120.61 (10)
Cl1—Sn1—Cl1^i^	180.0	N1—C6—C5	117.83 (10)
Cl3—Sn1—Cl2^i^	91.121 (10)	C7—C6—C5	121.56 (11)
Cl3^i^—Sn1—Cl2^i^	88.879 (10)	C10—C9—C8	120.70 (12)
Cl1—Sn1—Cl2^i^	90.704 (10)	C10—C9—H9	119.6
Cl1^i^—Sn1—Cl2^i^	89.295 (10)	C8—C9—H9	119.6
Cl3—Sn1—Cl2	88.879 (10)	C7—C8—C9	121.41 (12)
Cl3^i^—Sn1—Cl2	91.121 (10)	C7—C8—H8	119.3
Cl1—Sn1—Cl2	89.295 (10)	C9—C8—H8	119.3
Cl1^i^—Sn1—Cl2	90.706 (10)	C8—C7—C6	118.11 (11)
Cl2^i^—Sn1—Cl2	180.0	C8—C7—H7	120.9
C1—O1—H1	109.5	C6—C7—H7	120.9
H3A—O3—H3B	109.4	C4—C5—C10	123.20 (11)
C2—N1—C6	123.38 (10)	C4—C5—C6	118.68 (11)
C2—N1—H1A	118.5 (11)	C10—C5—C6	118.11 (11)
C6—N1—H1A	118.2 (11)	C9—C10—C5	120.10 (12)
N1—C2—C3	120.58 (10)	C9—C10—H10	120.0
N1—C2—C1	115.14 (10)	C5—C10—H10	120.0
C3—C2—C1	124.29 (10)	C3—C4—C5	120.63 (11)
O2—C1—O1	126.71 (11)	C3—C4—H4	119.7
O2—C1—C2	121.05 (10)	C5—C4—H4	119.7
			
C6—N1—C2—C3	1.99 (16)	N1—C6—C7—C8	−178.90 (11)
C6—N1—C2—C1	−177.71 (10)	C5—C6—C7—C8	0.58 (18)
N1—C2—C1—O2	−11.11 (16)	N1—C6—C5—C4	−1.35 (16)
C3—C2—C1—O2	169.20 (11)	C7—C6—C5—C4	179.15 (11)
N1—C2—C1—O1	168.63 (10)	N1—C6—C5—C10	178.41 (10)
C3—C2—C1—O1	−11.06 (16)	C7—C6—C5—C10	−1.09 (17)
N1—C2—C3—C4	−1.40 (17)	C8—C9—C10—C5	0.5 (2)
C1—C2—C3—C4	178.27 (11)	C4—C5—C10—C9	−179.70 (12)
C2—N1—C6—C7	178.92 (11)	C6—C5—C10—C9	0.55 (18)
C2—N1—C6—C5	−0.58 (16)	C2—C3—C4—C5	−0.54 (18)
C10—C9—C8—C7	−1.0 (2)	C10—C5—C4—C3	−177.86 (12)
C9—C8—C7—C6	0.47 (19)	C6—C5—C4—C3	1.89 (17)

Symmetry code: (i) −*x*, −*y*, −*z*.

### Bis(2-carboxyquinolinium) hexachloridostannate(IV) dihydrate Hydrogen-bond geometry (Å, º)

**Table:**

*D*—H···*A*	*D*—H	H···*A*	*D*···*A*	*D*—H···*A*
O1—H1···O3	0.82	1.75	2.5684 (14)	176
N1—H1*A*···O2^ii^	0.832 (17)	2.028 (17)	2.8348 (13)	163.4 (15)
O3—H3*A*···Cl1	0.85	2.46	3.2048 (11)	146
O3—H3*B*···Cl2^i^	0.85	2.61	3.3708 (11)	150
C3—H3···Cl1^iii^	0.93	2.97	3.6014 (12)	127
C7—H7···O2^ii^	0.93	2.58	3.2850 (15)	133
C7—H7···O3^ii^	0.93	2.51	3.3144 (16)	146

Symmetry codes: (i) −*x*, −*y*, −*z*; (ii) −*x*+2, −*y*+1, −*z*; (iii) −*x*+1, −*y*+1, −*z*+1.

## References

[bb1] Billing, D. G., Lemmerer, A. & Rademeyer, M. (2007). *Acta Cryst.* C**63**, m101–m104. 10.1107/S0108270107004970 17339700

[bb2] Bourhis, L. J., Dolomanov, O. V., Gildea, R. J., Howard, J. A. K. & Puschmann, H. (2015). *Acta Cryst.* A**71**, 59–75. 10.1107/S2053273314022207 PMC4283469 25537389

[bb3] Bruker (2013). *APEX2* and *SAINT*. Bruker AXS Inc., Madison, Wisconsin, USA.

[bb4] Dolomanov, O. V., Bourhis, L. J., Gildea, R. J., Howard, J. A. K. & Puschmann, H. (2009). *J. Appl. Cryst.* **42**, 339–341.

[bb5] Gelmboldt, V. O., Ganin, E. V. & Domasevitch, K. V. (2007). *Acta Cryst.* C**63**, o530–o534. 10.1107/S0108270107035469 17762125

[bb6] Ghallab, R., Boutebdja, M., Dénès, G. & Merazig, H. (2020). *Acta Cryst.* E**76**, 1279–1283. 10.1107/S205698902000941X PMC7405591 32844014

[bb7] Hajlaoui, S., Chaabane, I., Oueslati, A. & Guidara, K. (2013). *Solid State Sci.* **25**, 134–142.

[bb8] Krause, L., Herbst-Irmer, R., Sheldrick, G. M. & Stalke, D. (2015). *J. Appl. Cryst.* **48**, 3–10. 10.1107/S1600576714022985 PMC4453166 26089746

[bb9] Rademeyer, M. (2004). *Acta Cryst.* C**60**, m55–m56. 10.1107/S0108270103028270 14767112

[bb10] Sheldrick, G. M. (2015). *Acta Cryst.* C**71**, 3–8.

[bb11] Smith, G., Wermuth, U. D. & White, J. M. (2004). *Acta Cryst.* C**60**, o575–o581. 10.1107/S010827010401457X 15295192

[bb12] Smith, G., Wermuth, U. D. & White, J. M. (2008). *Acta Cryst.* C**64**, o180–o183. 10.1107/S0108270108004034 18322349

[bb13] Westrip, S. P. (2010). *J. Appl. Cryst.* **43**, 920–925.

